# Functional disability is related to serum chemerin levels in rheumatoid arthritis

**DOI:** 10.1038/s41598-021-87235-6

**Published:** 2021-04-16

**Authors:** Maria Luisa Vazquez-Villegas, Jorge I. Gamez-Nava, A. Miriam Saldaña-Cruz, Alfredo Celis, Esther N. Sanchez-Rodriguez, Edsaul Emilio Perez-Guerrero, Melissa Ramirez-Villafaña, Cesar Arturo Nava-Valdivia, Betsabe Contreras-Haro, Jose C. Vasquez-Jimenez, Juan M. Ponce-Guarneros, Ana K. Barocio-Ramirez, Sergio Cerpa-Cruz, Miriam F. Alcaraz-Lopez, Laura Gonzalez-Lopez

**Affiliations:** 1 Unidad de Medicina Familiar No., 4, Departamento de Epidemiologia, Instituto Mexicano del Seguro Social (IMSS), Guadalajara, Jalisco Mexico; 2 Programa de Doctorado and Coordinacion del Programa de Doctorado en Salud Publica, Departamento de Salud Publica, Instituto Regional de Investigacion en Salud Publica, Centro Universitario de Ciencias de la Salud, Universidad de Guadalajara, Guadalajara, Jalisco Mexico; 3 Programa de Doctorado en Farmacologia, Departamento de Fisiología, Instituto de Terapéutica Experimental y Clínica, Centro Universitario de Ciencias de la Salud, Universidad de Guadalajara, Guadalajara, Mexico; 4 Unidad de Investigación Biomédica 02, UMAE, Hospital de Especialidades, Centro Médico Nacional de Occidente, IMSS, Guadalajara, Mexico; 5 Instituto de Investigación en Ciencias Biomédicas, Centro Universitario de Ciencias de la Salud, Universidad de Guadalajara, Guadalajara, Mexico; 6 Departamento de Microbiología y Patología, Centro Universitario de Ciencias de La Salud, Universidad de Guadalajara, Guadalajara, Mexico; 7 Departamento de Ciencias Biomedicas, Centro Universitario de Tonalá, Universidad de Guadalajara, Guadalajara, Mexico; 8 Centro Universitario de Investigaciones Biomédicas, Universidad de Colima (UCOL), Colima, Mexico; 9 Division de Reumatologia, Hospital Civil de Guadalajara “Fray Antonio Alcalde”, Guadalajara, Jalisco Mexico; 10 Servicio de Medicina Interna-Reumatología, Hospital General Regional 46, IMSS, Guadalajara, Jalisco Mexico; 11 Servicio de Medicina Interna-Reumatología, Hospital General Regional 110, IMSS, Guadalajara, Jalisco Mexico

**Keywords:** Rheumatic diseases, Biomarkers, Inflammation

## Abstract

Adipokines, especially chemerin, can interact with cytokines and other molecules in inflammation. To date, there is insufficient information regarding a possible correlation between functional disability and chemerin and other pro-inflammatory molecules in rheumatoid arthritis (RA). To identify the association of functional disability with serum chemerin and other pro-inflammatory molecules, including other adipokines, cytokines and E-selectin, in patients with RA. Cross-sectional study. Assessment: disease activity (DAS28-ESR) and functional disability (HAQ-DI). We compared the adipokines (chemerin, leptin, adiponectin, resistin, and visfatin), cytokines (TNF-α, IL-6, IL-1β, and IL-18) and E-selectin levels between RA with functional disability and RA non-disabled patients. Of 82 patients with RA, 43 (52%) had functional disability. The RA with functional disability group had higher chemerin (140 vs. 112 ng/mL, p = 0.007) than the non-disabled RA group. Chemerin correlated with the HAQ-DI (rho = 0.27, p = 0.02) and DAS28-ESR (rho = 0.21, p = 0.05). Severe activity correlated with IL-6 (rho = 0.33, p = 0.003) and E-selectin (rho = 0.23, p = 0.03) but not with disability. No other pro-inflammatory molecules correlated with HAQ-DI. High chemerin levels were associated with functional disability in RA, whereas no other molecules correlated with loss of function. These results encourage further studies assessing new roles of chemerin as a marker of impairment in RA.

## Introduction

Rheumatoid arthritis (RA) is a disorder characterized by chronic inflammatory involvement of the synovial joints leading to cartilage destruction, intra-articular bone erosion, joint subluxation and bone ankylosis^1^. Many patients with RA with bone erosions develop progressive radiological destruction and disability^2,3^. Functional disability is an important outcome measure associated with deteriorated health-related quality of life. Avoiding functional disability is an important objective for therapy in these patients. Many serum markers of disease activity have been assessed in the context of RA, such as the erythrocyte sedimentation rate, C-reactive protein, some pro-inflammatory cytokines and, most recently, the levels of certain adipokines^2,4^. Adipokines are hormone-like molecules synthesized by adipose tissue that can have several effects on the endocrine, cardiovascular and immune systems^5,6^. However, these markers do not have a consistent association with functional disability.

Previous studies performed with patients with RA have revealed abnormally increased levels of adipokines, including leptin, resistin, adiponectin, and visfatin, in comparison with the respective levels observed in controls^5,7–9^. Some works performed in patients with RA have revealed that serum chemerin increases during disease activity episodes. Ha et al., comparing active versus inactive RA, identified increased chemerin levels in active disease^10^. Tolusso et al. also observed that chemerin levels increase with the severity of disease activity^11^.

The effect of chemerin on inflammation in RA is supported by findings observed in experimental studies. Kaneko et al., in an experimental study, observed that fibroblast-like synoviocyte (FLSs) incubated with chemerin had enhanced production of IL-6, chemokine (C–C motif) ligand 2 (CCL2) and metalloproteinase 3 (MMP-3)^12^. These authors identified that FLSs incubated with chemerin increased the motility of the FLSs, augmented the phosphorylation of p38MAPK and enhanced the phosphorylation of Akt^12^.

These findings indicate that chemerin can be considered a marker of disease activity. Nevertheless, to date, only a few works have suggested that an increase in the levels of chemerin is related to functional impairment in patients with RA^11,13^. Tolusso et al. identified a relationship between serum chemerin and disability assessed by a health-assessment questionnaire in early RA^11^. Makrilaki et al. assessed 19 patients with RA receiving treatment with tocilizumab for six months and identified a correlation between baseline chemerin levels and the health-assessment questionnaire disability index (HAQ-Di) score at baseline and at six months of tocilizumab treatment^13^.

These two previous studies did not evaluate the possible interactions of chemerin with other molecules, including other adipokines, cytokines and E-selectin, that could also be associated with functional disability. Of these molecules, E-selectin is an adhesion molecule upregulated at inflammation and matrix destruction sites in RA^14^. This adhesion molecule participates in cell-to-cell adhesion in inflamed synovium^15^. High levels of E-selectin are potential markers of worse disease evolution in patients with RA. E-selectin levels were associated with disease activity, including swollen joint count and C-reactive protein levels; moreover, these E-selectin levels were associated with the progression of RA and functional disability score at five years^16^. Our group has also reported that high levels of E-selectin correlated with tender joint count, morning stiffness, severity of pain, disease activity and high disability index scores in a group of patients with RA^17^. Interestingly, one experimental model described that some adipokines can enhance the interaction between synovial fibroblasts and endothelial cells, whereas glucocorticoids can decrease this process^18^. Therefore, new studies assessing the role of adipokines, cytokines and E-selectin as potential markers of functional disability in patients with RA are still required.

Thus, the objective of the present study was to identify the association of functional disability with the serum levels of chemerin and several pro-inflammatory molecules, such as other adipokines (leptin, adiponectin, resistin and visfatin), cytokines (TNF-α, IL-6, IL-1β and IL-18) and E-selectin.

## Results

We included 82 women with RA. Forty-three (52%) patients had some grade of functional disability.

Table 1 shows the comparison of characteristics between patients with RA with functional disability and patients with RA without functional disability. These two groups had similar ages and disease durations. The patients with RA with functional disability had a higher mean body mass index than patients without functional disability (28 versus 26, p = 0.03). Serum chemerin was significantly elevated in patients with RA with functional disability compared with the levels in RA patients without disability (median 140 versus 112 ng/mL, p = 0.007). RA patients with deteriorated functioning had a higher frequency of corticosteroid therapy (76% versus 49%, p = 0.01).

**Table 1:** Comparison of clinical and laboratory characteristics between rheumatoid arthritis with functional disability (RA with functional disability) versus rheumatoid arthritis without disability (RA without disability).

Variables	RA with functional disability (n = 43)	RA without disability (n = 39)	*p*
Age (years)	56 (35–77)	59 (30–79)	0.67
Body mass index	28 (19–35)	26 (19–46)	***0.03***
Disease duration (years)	10 (0.1–30)	9 (0.1–30)	0.40
History of fractures	13 (30)	11(28)	0.84
RF (IU/mL)	57 (8–765)	20 (5–118)	0.07
ESR (mm/h)	29 (5–354)	22 (0.1–77)	0.07
DAS28-ESR	3.4 (2.1–7.1)	2.5 (1.6–4.4)	** < 0.001**
**Adipokines**			
Chemerin, (ng/mL)	140 (71–1402)	112 (56–277)	***0.007***
Leptin, (ng/mL)	30 (3–175)	33 (7–99)	0.87
Adiponectin, (ng/mL)	10,939 (1328–25,529)	8927 (1368–24,532)	0.83
Resistin, (pg/mL)	7 (2–13)	6 (4–20)	0.13
Visfatin, (ng/mL)	20 (2–255)	20 (2–278)	0.90
**Cytokines**			
IL-6, (pg/mL)	6 (0.7–110)	4 (0.4–29)	0.13
TNF-α, (pg/mL)	6 (0.5–714)	5 (0.3–668)	0.09
IL-1β, (pg/mL)	7 (1–11)	7 (5–15)	0.88
IL-18, (pg/mL)	370 (137–1027)	388 (127–1767)	0.51
**Adhesion cell molecule**			
E-Selectin, (pg/mL)	46 (9–84)	40 (13–84)	0.09
**Treatment**			
Methotrexate, n (%)	20 (48)	26 (67)	0.08
Sulfasalazine, n (%)	20 (48)	17 (44)	0.71
Chloroquine, n (%)	9 (21)	11 (28)	0.48
Azathioprine, n (%)	7 (17)	3 (8)	0.31
b-DMARDs, n (%)	7 (17)	4 (10)	0.52
Corticosteroids therapy, n (%)	32 (76)	19 (49)	***0.01***
Prednisone doses, mg/day	5 (2.5–10)	5 (2.5–10)	0.93

*RF* rheumatoid factor, ERS erythrocyte sedimentation rate, *IL-6* interleukin-6, *TNF-α* tumor necrosis factor α, *IL-1β* interleukin-Beta 1, *IL-18* interleukin-18, *b-DMARDs* biologic-disease modifying antirheumatic drugs.

(A) Rheumatoid arthritis with functional disability (HAQ-Di, score ≥ 0.6); and (B) rheumatoid arthritis without functional disability (HAQ-Di, score < 0.6). Qualitative variables are expressed in medians (ranges). Proportions were compared with chi square test and medians were compared with Mann Whitney U test.

In the comparison of patients with RA with functional disability versus patients with RA without functional impairment, there were no differences observed in other adipokines (adiponectin, leptin, resistin and visfatin), cytokines (IL-6, TNF-α, IL-1β and IL-18), E-selectin, rheumatoid factor, or ESR.

In the subsection of data availability for more information, we compared RA patients with Steinbrocker staging radiological score III or IV versus RA patients with a Steinbrocker staging radiological score I or II, the presence of functional disability was higher with those with Steinbrocker III or IV (n = 21 (49%) versus n = 9 (23%), p = 0.016). There were no differences observed in the medians of chemerin levels [133 (Ranges: 75–277) versus 130 (Ranges: 56–1402), p = 0.41] and frequency of corticosteroids therapy (n = 20 (67%) versus n = 35 (67%) p = 0.87).

In the supplementary Fig 1. we show the comparison between RA patients with corticosteroids therapy versus RA patients without corticosteroids therapy (supplementary Fig. 1A). The patients with corticosteroids therapy had a trend for higher chemerin levels than patients without corticosteroids therapy that not achieved statistical significance [137 (Ranges: 56–1402) versus 117 (Ranges: 73–216), p = 0.052]. Additionally, in the supplementary Fig. 1 is shown the comparison between RA patients with BMI ≥ 25 versus RA patients with BMI < 25. We do not observe differences of chemerin levels [128 (Ranges: 71–1402) versus 131 (Ranges: 56–235), p = 0.8] (supplementary Fig. 1B).

Table 2 shows the correlations of the functional score (HAQ-Di) and disease activity score (DAS28-ESR) with serum chemerin and serum levels of other adipokines (adiponectin, leptin, resistin and visfatin), cytokines (IL-6, TNF-α, IL-1β and IL-18) and E-selectin levels.

**Table 2:** Correlations of the functional score (HAQ-Di) and disease activity score (DAS28-ESR) with the serum chemerin, the serum levels of other adipokines (adiponectin, leptin, resistin and visfatin), cytokines (IL-6, TNF-α, IL-1β and IL-18) and E-selectin levels in Rheumatoid Arthritis patients.

Rheumatoid arthritis, n = 82	HAQ-Di		DAS 28-ESR	
	Rho	*p*	Rho	*p*
**Adipokines**				
Chemerin, (ng/mL)	***0.27***	***0.02***	***0.21***	***0.05***
Adiponectin, (ng/mL)	− 0.02	0.85	− 0.13	0.23
Leptin, (ng/mL)	0.06	0.68	0.13	0.37
Resistin, (pg/mL)	0.17	0.13	0.11	0.32
Visfatin, (ng/mL)	− 0.06	0.60	0.12	0.29
**Cytokines**				
IL-6, (pg/mL)	0.18	0.11	***0.33***	***0.003***
TNF-α , (pg/mL)	0.14	0.21	0.11	0.31
IL-1β, (pg/mL)	0.05	0.64	0.01	0.90
IL-18, (pg/mL)	− 0.04	0.70	0.11	0.32
**Adhesion cell molecule**				
E-Selectin, (pg/mL)	0.15	0.17	***0.23***	***0.03***

Spearman correlation test (rho).

*IL-6* interleukin-6, *TNF-α* tumor necrosis factor α, *IL-1β* interleukin-Beta 1, *IL-18* interleukin-18.

Supplementary Fig. 2 shows that elevated HAQ-Di scores correlated significantly with increased chemerin (rho 0.27, p = 0.02) (supplementary Fig. 2A); and a correlation between higher disease activity with the increased serum chemerin concentration (rho 0.21, p = 0.05) (supplementary Fig. 2B). Additionally, in the supplementary Fig. 2 is shown that the DAS28-ESR score correlated with elevated IL-6 levels (rho 0.33, p = 0.003) (supplementary Fig. 2C), and high E-selectin levels (rho 0.23, p = 0.03) (supplementary Fig. 2D). No other correlations were observed between HAQ-Di or DAS28-ESR and adipokines (adiponectin, leptin, resistin and visfatin) or other cytokines (TNF-α, IL-1β and IL-18).

Table 3 shows the sub-analysis of the comparisons between three subgroups: (a) patients with RA with functional disability (HAQ-Di, score ≥ 0.6) and moderate or severe disease activity (DAS28-ESR > 3.2); (b) patients with RA with functional disability (HAQ-Di, score ≥ 0.6) and low disease activity or remission (DAS28-ESR ≤ 3.2); and c) patients with RA without functional disability. These groups were similar in age, disease duration and rheumatoid factor levels. Serum chemerin was higher in the subgroup of patients with RA with functional disability and DAS28-ESR > 3.2 than in the subgroup of patients with RA without disability (p = 0.008). There were no trends towards differences in the serum levels of the other adipokines, cytokines and E-selectin among the three groups.

**Table 3:** Comparison of clinical and laboratory characteristics between three groups according functional disability and disease activity.

Variables	Disability + high DAS28-ESR (n = 24)	Disability + low DAS28-ESR (n = 19)	Non-disabled (n = 39)	*p*
Age (years)	56 (35–73)	55 (37–77)	59 (30–79)	0.90
Body mass index	28 (19–35)	28 (22–34)	26 (19–46)	0.10
Disease duration (years)	14 (0.1–27)	8 (1–30)	9 (0.1–30)	0.39
RF (IU/mL)	57 (8–765)	67 (9–641)	20 (5–118)	0.18
ESR (mm/h)	33.5 (5–354)	24 (7–45)	21 (0.1–43)	0.06
**Adipokines**				
Chemerin, (ng/mL)	160 (77–1402)	136 (71–235)	112 (56–277)	***0.008***
Leptin, (ng/mL)	31 (3–115)	25 (4–175)	33 (7–99)	*0.98*
Adiponectin, (ng/mL)	7767 (1328–25,529)	12,886 (3921–17,166)	8927 (1368–24,532)	0.33
Resistin, (pg/mL)	7 (2–13)	7 (4–10)	6 (4–20)	0.29
Visfatin, (ng/mL)	19 (2–172)	21 (6–255)	20 (2–278)	0.87
**Cytokines**				
IL-6, (pg/mL)	7 (1–110)	6 (1–32)	4 (0.4–29)	0.10
TNF-α , (pg/mL)	6 (1–474)	6 (3–714)	5 (0.3–668)	0.18
IL-1β, (pg/mL)	7 (5–11)	7 (1–9)	7 (5–15)	0.87
IL-18, (pg/mL)	389 (137–1027)	333 (141–672)	388 (127–1767)	0.60
**Adhesion cell molecule**				
E-Selectin, (pg/mL)	48 (32–84)	39 (9–84)	40 (13–84)	0.12
**Treatment**				
Methotrexate, n (%)	11 (46)	9 (47)	26 (67)	0.22
Sulfasalazine, n (%)	9 (38)	11 (58)	17 (44)	0.30
Chloroquine, n (%)	6 (25)	3 (16)	11 (28)	0.64
Azathioprine, n (%)	4 (17)	3 (16)	3 (8)	0.47
b-DMARDs, n (%)	6 (25)	3 (16)	4 (10)	0.13
Corticosteroid, n (%)	21 (88)	18 (95)	19 (49)	***0.008***
Prednisone doses, mg/day	5 (2.5–10)	5(2.5–7.5)	5 (2.5–10)	0.30

*RF* rheumatoid factor, *ESR* erythrocyte sedimentation rate, *IL-6* interleukin-6, *TNF-α* tumor necrosis factor α, *IL-1β* interleukin-Beta 1, *IL-18* interleukin-18.

Three groups: (a) rheumatoid arthritis with functional disability and higher disease activity (disability: HAQ-Di, score ≥ 0.6 + high DAS28-ESR, score > 3.2); (b) rheumatoid arthritis with functional disability and lower disease activity (disability: HAQ-Di, score ≥ 0.6 + low DAS28-ESR, score ≤ 3.2) and (c) rheumatoid arthritis patients without functional disability (non-disabled). Qualitative variables are expressed in frequency (%); quantitative variables are expressed in median (ranges). Proportions were compared with chi square test and the comparison of quantitative variables was performed with Kruskal–Wallis test.

Regarding the data that are not shown in the tables, we investigated possible correlations between the levels of chemerin, other adipokines (adiponectin, leptin, resistin and visfatin), cytokines (IL-6, TNF-α, IL-1β and IL-18) and E-selectin in patients with RA. The levels of chemerin were positively correlated with TNF-α (rho 0.24, p = 0.03) and negatively correlated with the levels of leptin (rho − 0.23, p = 0.04). The adiponectin level was positively correlated with IL-1β (rho 0.26, p = 0.02). The leptin concentration correlated with visfatin (rho 0.27, p = 0.01) and E-selectin (rho 0.28, p = 0.009). E-selectin correlated with IL-6 (rho 0.31, p = 0.005).

## Discussion

This work shows that high chemerin concentrations were significantly related to functional disability in patients with RA. The chemerin level was correlated with functional disability and disease activity score. This association of chemerin was independent of other adipokines, cytokines, and E-selectin. The other adipokines evaluated were not related to functional disability.

Chemerin is a chemokine produced by adipose tissue cells, mainly in visceral adipose tissue. Chemerin is an inducer of adipocyte differentiation and increases metabolism and adipogenesis^19^. Chemerin is described as a pro-inflammatory adipokine participating in the inflammatory processes of chronic diseases. In vitro, chemerin stimulates the production of several cytokines, including IL-6, TNF-α, and IL-1β, and participates in the synthesis of metalloproteinases (MMPs) by stimulating chondrocytes and some chemokines^19–23^. Chemerin receptor 23 is expressed on several cells involved in the immune response, including macrophages, dendritic cells, and NK lymphocytes. These findings support that changes in chemerin levels can participate in the coactivation of some cells of the inflammatory response^23–25^. In RA, the expression of chemerin is also increased on FLSs^12^. We observed a relationship between chemerin and increased serum levels of TNF-α. This finding is explained by the fact that the synthesis of chemerin can be enhanced by pro-inflammatory cytokines, including TNF-α^19,26^. According to our findings, high serum chemerin in patients with RA was associated with an impairment in functional capacity and with active disease. These findings have been supported by other groups: Makrilakis et al. observed in 19 patients with RA who were undergoing treatment with tocilizumab for 6 months that baseline chemerin level was correlated with the HAQ-Di score^13^. Tolusso et al. investigated patients with early RA who were included in a treatment-to-target strategy and a group of overweight/obese patients with RA^11^. These authors identified a correlation between chemerin and HAQ-Di scores.

Regarding the relationship between chemerin levels and the severity of disease activity identified in our study, these findings are supported by other authors. Ha et al. identified higher levels of chemerin in patients with RA patients with disease activity^10^. Nevertheless, these findings were not observed by other authors. Fioravanti et al. did not find a correlation between chemerin levels and the DAS28-ESR score; in their study, among patients with RA treated with tocilizumab monotherapy or tocilizumab combined with methotrexate, a correlation was not observed, and none of the patients had a history of other treatments^27^.

### Strengths of the study

This study identified an association between functional disability and chemerin levels in patients with RA with long disease durations treated with synthetic or biologic DMARDs. One of the strengths was that our study performed a comprehensive assessment of pro-inflammatory molecules, including adipokines, cytokines and E-selectin, that have been involved in the inflammatory processes in patients with RA. Our findings support that chemerin is associated with increased functional disability independent of other adipokines. Chemerin was also associated with more active disease. These findings support the importance of investigating the presence of high chemerin levels as a marker of functional impairment in patients with RA. Additionally, this study has a sample size that allows us to test associations with sufficient statistical power to minimize a possible type II error.

### Limitations of the study

Our study has some limitations. Chemerin, other adipokines, cytokines and E-selectin were determined only at a single point in the time; therefore, we do not have information about the levels of the serum molecules in patients who were not treated at the onset. Future studies should be performed in early RA without treatment to identify if the treatment could change the levels of these molecules. To assess the generalizability of our results, we included only female patients in the study; there are differences in the serum levels of adipokines between women and men. Therefore, we must be cautious in extrapolating these results to men with RA. Future studies should include a group of men with RA to evaluate whether sex is a confounder in the association observed between chemerin and functional disability.

Another limitation is the possibility that these findings can be explained by chance. This possibility should always be considered, especially when these findings are observed in a first study, although we have found a significant association between functional disability and high levels of chemerin and a correlation between the serum chemerin levels and high scores of functional disability. Both of these findings must be observed by other future studies to demonstrate the consistency of our findings.

Therefore, this design prevented the identification of a possible causal relationship, and we can only hypothesize that chemerin levels contribute to the damage leading to functional impairment in patients with RA.

This limitation reflects the requirement for longitudinal studies to assess whether high levels of chemerin predict the progression of functional deterioration.

## Conclusions

This study identified an association between higher chemerin levels and functional disability in patients with RA. This association was not observed with the remaining pro-inflammatory molecules, including other adipokines, cytokines and E-selectin. These results encourage further studies assessing new disease features in patients with RA with increased chemerin levels. Longitudinal studies are needed to determine the effects of persistently increased chemerin levels on progressive functional impairment in patients with RA.

## Methods

### Design: cross-sectional study

#### Setting

Patients with RA attending an outpatient rheumatology consultation at a secondary-care centre in Guadalajara, Mexico.

### Inclusion and exclusion criteria

The inclusion criteria were females aged ≥ 18 years with RA diagnosed by a rheumatologist on the basis of the ACR 1987 criteria for RA. The rationality of including only women for this study was the variation in the serum levels of adipokines between women and men that can introduce a potential confounder. Therefore, in this study, we decided to exclude men with RA. Other exclusion criteria were pregnancy, lactation, active infection, diabetes mellitus, the presence of thyroid disease, a blood transfusion in the previous 3 months or a diagnosis of overlap syndrome.

### Clinical evaluation

Two trained researchers completed a structured clinical chart containing epidemiological and clinical variables and treatments and performed a physical examination. The assessment of functional disability was performed using the Spanish version of the Health Assessment Questionnaire-Disability Index (HAQ-Di)^28^. We chose the cut-off point of a HAQ-Di score ≥ 0.6 to classify a patient as having functional disability. Patients with a HAQ-Di score < 0.6 were considered to be “without disability”. The activity of the disease was investigated using DAS28-ESR. The erythrocyte sedimentation rate (ESR, mm/h) was measured with Westergren, and C-reactive protein levels (CRP, mg/mL) and rheumatoid factor titres (RF, IU/mL) were quantified by nephelometry.

To categorize the severity of disease activity, we used the cut-off point of a DAS28-ESR score > 3.2 to classify a patient with “moderate or high activity disease”, and patients with a DAS28-ESR score ≤ 3.2 were considered to have “low disease activity or in remission”^29^. Body mass index (BMI) (weight in kg/square of height measured in metres) was recorded.

### Radiological damage

We used the Steinbrocker system for staging the radiological damage of RA on plain radiographs of hands. This system was categorized in: Grade I—periarticular osteopenia without destructive changes, Grade II—erosions present with or without joint space narrowing, Grade III—articular subluxations, Grade IV—bone ankylosis^30^.

### Quantification of adipokines, cytokines and E-selectin

The patients’ adipokine levels (chemerin, leptin, adiponectin, resistin and visfatin) were assessed in serum obtained after > 12 h of fasting. Serum adipokine concentrations were quantified by ELISA (Quantikine Human Immunoassay; R&D Systems, Minneapolis, MN, USA). Leptin sensitivity was 7.8 pg/mL, adiponectin sensitivity was 0.08 ng/mL, chemerin sensitivity was 1.08 pg/mL, resistin sensitivity was 0.01 ng/mL, and visfatin sensitivity was 1 ng/mL (Mybiosource San Diego California USA).

Additionally, serum pro-inflammatory cytokines (TNF-α, IL-6, IL-1β and IL-18) were measured by ELISA using commercial kits. TNF-α sensitivity was 0.5 pg/mL, IL-6 sensitivity was 0.7 pg/mL, and IL-1β sensitivity was 1 pg/mL (R&D Systems, Minneapolis, MN, USA). For IL-18, the sensitivity was 12.5 pg/mL (MBL International).

The assay for the adhesion molecule E-selectin had a sensitivity of 0.009 ng/mL (R&D Systems, Minneapolis MN USA). All procedures for the measurement of these molecules were conducted by researchers blinded to the patients’ clinical characteristics.

### Statistical analysis

We hypothesized that chemerin levels are increased in patients with RA with clinical disability in comparison with patients with RA without clinical disability, and this increase is independent of other adipokines, cytokines or E-selectin. To test this hypothesis, we planned a correlation analysis between two continuous variables; the first was adipokines as independent variables and DAS28-CRP or HAQ-DI scores as the dependent variables. Using the results described in a previous study performed by Ha et al.^10^, these authors observed that chemerin levels correlated with the DAS-28 score in patients with RA (r = 0.31, p < 0.01).

Thus, we proceeded to assume the following:

1. A result of the Spearman correlation tests of 0.31, with a significant p-value, could be sufficient to reject the null hypothesis of no correlations between the markers assessed in this study and the HAQ-DI score (functional disability).
2. We chose a statistical power of 0.8 and a type I error probability of 0.05.

Thus, we found that 80 patients with RA were estimated to be required to reject the null hypothesis of no correlations between these markers and the HAQ-DI score.

Quantitative variables are described by medians and ranges, and qualitative variables are described by frequencies (%). According to the Shapiro–Wilk test, the adipokines, cytokines and E-selectin levels showed a nonparametric distribution. Therefore, we assessed the differences in the medians of adipokines, cytokines and E-selectin levels between patients with RA with functional disability and patients with RA without functional disability using the Mann–Whitney U test. The chi-square test was used to demonstrate differences between proportions. Spearman’s tests were utilized to determine the correlations of the HAQ-Di and DAS28-ESR with chemerin, other adipokines, pro-inflammatory cytokines and E-selectin.

The Kruskal–Wallis test was utilized for comparisons of the sum of ranks between three groups: (a) patients with functional disability and disease activity (HAQ-Di, score ≥ 0.6 + High DAS28-ESR, score > 3.2); (b) patients with functional disability without disease activity (HAQ-Di, score < 0.6 + Low DAS28-ESR, score ≤ 3.2); and (c) patients without functional disability (non-disabled).

The cut-off for statistical significance was p ≤ 0.05. Statistical analyses were performed using R version 4.0.0.

### Ethics declarations

This study protocol was approved by the Research Ethical Committee of the Instituto Mexicano del Seguro Social, Jalisco, Mexico. Approval code: R-2009-1303-13. Each procedure conducted during the study was performed following the guidelines of the Declaration of Helsinki. All participants signed a voluntary informed consent prior to study onset.

### Consent for publication

All the authors declare their consent for publication.

## Supplementary Information

Supplementary Information.

## Data Availability

The datasets generated and/or analysed during the current study are available from the corresponding author on reasonable request.
