# Supplementary material for: Functional disability is related to serum chemerin levels in rheumatoid arthritis
Source: Sci Rep. 2021 Apr 16;11:8360. doi: 10.1038/s41598-021-87235-6 (PMC8052358; doi:10.1038/s41598-021-87235-6)
Supplement: Supplementary file 1 — Supplementary Information. [file 41598_2021_87235_MOESM1_ESM.docx]

Complementary Table 1. Comparison of clinical and laboratory characteristics between rheumatoid arthritis with Steinbrocker III or IV stages score versus rheumatoid arthritis with Steinbrocker I or II stages score

| Variables | III or IV stages  n= 30 | I or II stages  n=52 | *p* |
| --- | --- | --- | --- |
| Functional Disability, n (%) | 21 (70) | 22 (42) | ***0.016*** |
| Corticosteroids therapy, n (%) | 20 (67) | 35 (67) | 0.87 |
| Chemerin, (ng/mL) | 133 (75-277) | 130 (56-1402) | 0.41 |

Qualitative variables are expressed in medians (ranges). Proportions were compared with chi square test and medians were compared with Mann Whitney U test.

Complementary Table 2. Comparison of chemerin levels between rheumatoid arthritis with corticosteroids therapy versus rheumatoid arthritis without corticosteroids therapy.

| Variable | Corticosteroids therapy n= 51 | Without Corticosteroids therapy n=30 | *p* |
| --- | --- | --- | --- |
| Chemerin, (ng/mL) | 137 (56-1402) | 117 (73-216) | ***0.052*** |

Qualitative variables are expressed in medians (ranges) and medians were compared with Mann Whitney U test.

Complementary Table 3. Comparison of chemerin levels between rheumatoid arthritis with body mass index ≥ 25 versus rheumatoid arthritis with body mass index <25.

| Variable | BMI≥ 25  n= 53 | BMI<25  n=27 | *p* |
| --- | --- | --- | --- |
| Chemerin, (ng/mL) | 128 (71-1402) | 131 (56-235) | 0.81 |

BMI: body mass index. Qualitative variables are expressed in medians (ranges) and medians were compared with Mann Whitney U test.


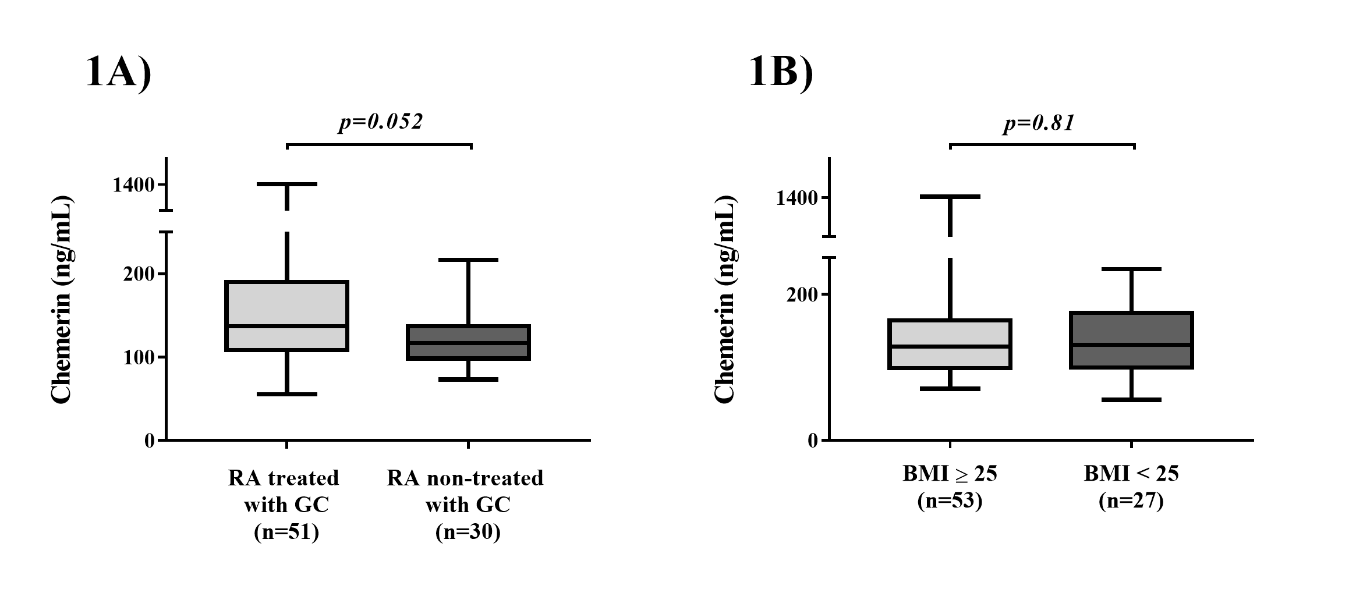


**Supplementary Figure 1.** **Comparison of serum chemerin levels between use of glucocorticoids therapy and body mass index (BMI) in RA patients.** *p-values* were obtained using Mann-Whitney U test. Figure 1A shows the comparison of serum chemerin levels between corticosteroid therapy and without corticosteroids therapy groups. Figure 1B shows the comparison of serum chemerin levels between patients with body mass index ≥ 25 and body mass index < 25.


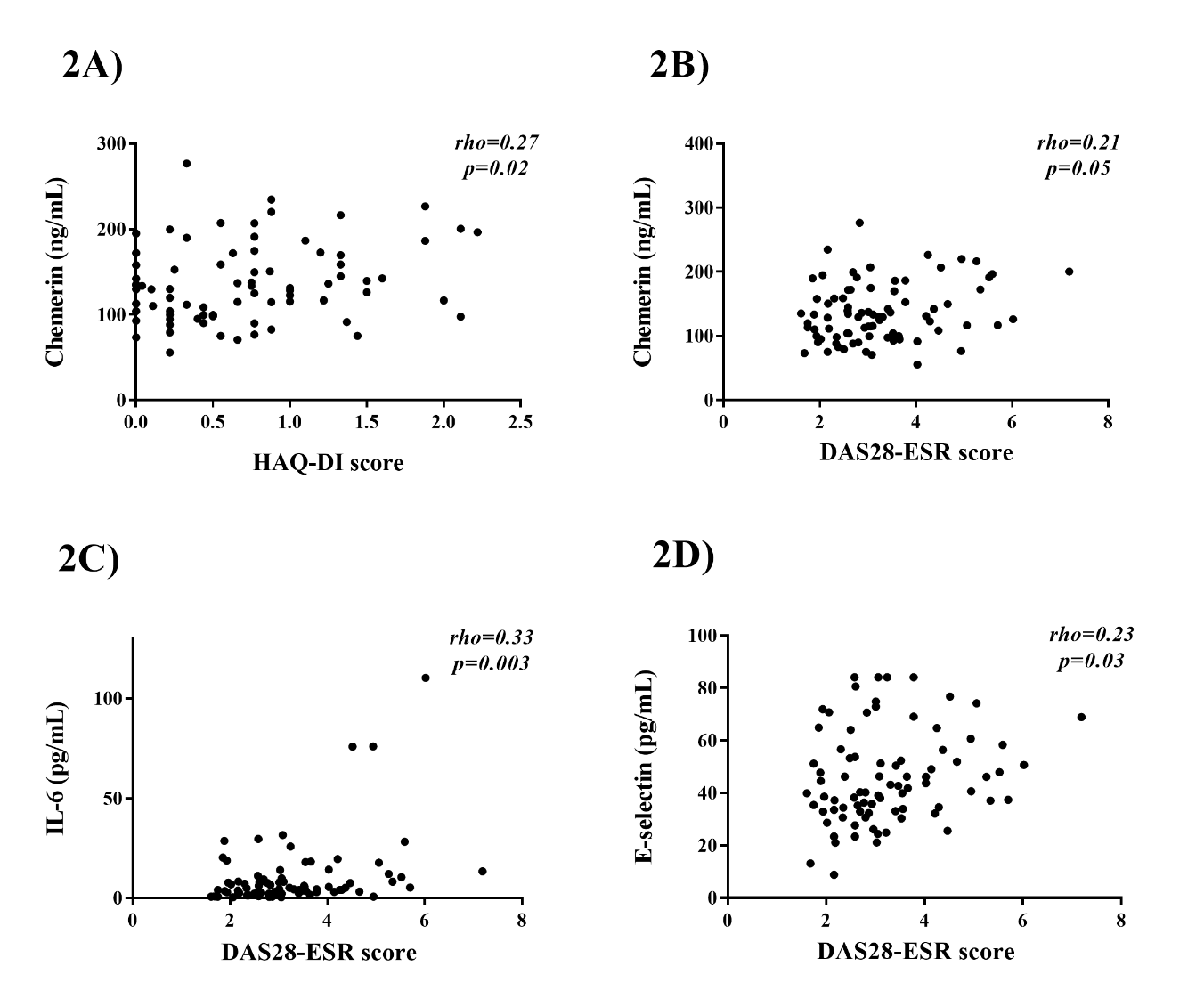


**Supplementary Figure 2. Correlation between DAS28-ESR score and HAQ-DI score with serum chemerin, IL-6 and E-selectin levels in RA patients.** *Rho-value* and *p-values* were obtained using Spearman’s rank correlation coefficient. Figure 2A shows the correlation between HAQ-DI score and serum chemerin levels. Figure 2B shows the correlation between DAS28-ESR score and serum chemerin levels. Figure 2C shows the correlation between DAS28-ESR score and serum IL-6 levels. Figure 2D shows the correlation between DAS28-ESR score and E-selectin levels.
